# Prognostic Value of the Pretreatment Neutrophil-to-Lymphocyte Ratio in Pediatric Parotid Cancer

**DOI:** 10.3389/fped.2019.00207

**Published:** 2019-05-24

**Authors:** Dongjie Seng, Qigen Fang, Peng Li, Fei Liu, Shanting Liu

**Affiliations:** ^1^Department of Otorhinolaryngology, The Affiliated Children Hospital of Zhengzhou University, Henan Children Hospital, Zhengzhou, China; ^2^Department of Head Neck and Thyroid, Affiliated Cancer Hospital of Zhengzhou University, Henan Cancer Hospital, Zhengzhou, China; ^3^Department of Oral Medicine, The First Affiliated Hospital of Zhengzhou University, Zhengzhou, China

**Keywords:** salivary gland cancer, parotid cancer, prognosis, pediatric cancer, neutrophil-to-lymphocyte ratio

## Abstract

**Objective:** Our goal was to evaluate the prognostic significance of the neutrophil-to-lymphocyte ratio (NLR) in pediatric patients with parotid cancer.

**Materials and Methods:** Pediatric patients with primary parotid cancer were retrospectively enrolled from several clinical centers. The associations between the clinical-pathologic variables and NLR and the prognostic significance of NLR for recurrence-free survival (RFS) and disease-specific survival (DSS) were analyzed.

**Results:** A total of 123 patients were included. The mean NLR was 2.51 and ranged from 1.7 to 6.1. The tumor stage and disease grade were significantly related to NLR. In patients with NLR < 2.51, the 10-year RFS rate was 97%, and in patients with NLR ≥ 2.51, the 10-year RFS rate was 84%; the difference was significant (*p* = 0.016). In patients with NLR < 2.51, the 10-year DSS rate was 98%, and in patients with NLR ≥ 2.51, the 10-year DSS rate was 83%; this difference was also significant (*p* = 0.035). Further Cox model analysis confirmed the independence of NLR in predicting the RFS and DSS rates.

**Conclusions:** NLR is significantly associated with prognosis in pediatric patients with parotid cancer.

## Introduction

Parotid tumors are uncommon in children and adolescents; however, 10–50% of these tumors are malignant (1–3); because of the rarity of the disease, it is challenging to develop a consensus regarding treatment. Moreover, clinical-pathologic characteristics and prognosis show differences between pediatric and adult patients with parotid cancer (4); it was reported that prognostic factors in pediatric patients included high tumor stage, high histologic grade, perineural invasion, and lymphovascular invasion (5–9).

Interactions between the tumor microenvironment and tumor cells play an important role in cancer progression, and the microenvironment includes metabolic, inflammatory, and immune responses to stimuli from the surrounding tissue. A number of authors have previously indicated that the systemic inflammatory response could promote tumor metastasis, microvascular regeneration, and tumor cell proliferation (10–12); further, the peripheral neutrophil-to-lymphocyte ratio (NLR) is a reliable and accurate inflammatory marker. High NLR is thought to be significantly associated with worse survival in solid cancers (13, 14), including head and neck squamous cell carcinoma, breast cancer, and prostate cancer. Nevertheless, the significance of NLR in parotid cancer remains unknown. Therefore, considering the immature lymphatic defense system in children, our goal was to analyze the prognostic value of NLR in pediatric patients with parotid cancer.

## Materials and Methods

The Zhengzhou University institutional research committee approved our study (No. FHN2017127), and all legal guardians, including parents, provided written informed consent for any patient under the age of 18; this study was conducted in accordance with the Declaration of Helsinki.

Between January 1995 and December 2016, pediatric patients (≤18 years old) undergoing surgery for primary parotid cancer were retrospectively enrolled from three hospitals: Affiliated Cancer Hospital, Affiliated Pediatric Hospital and The First Affiliated Hospital of Zhengzhou University. Data, including age, sex, TNM stage (according to AJCC 2017), disease grade, intraparotid node metastasis (IPN), surgery, pathologic report, and follow-up, were extracted and analyzed. All pathologic sections were re-reviewed by at least two pathologists.

NLR was defined as absolute neutrophil count divided by absolute lymphocyte count within 2 weeks of initial treatment (13–16). The cutoff values were calculated from ROC curves, means, tertiles, or medians from previous studies and varied from 1.98 to 5 (13–17). The standard cutoff value remains unknown; in the present study, the cutoff value was defined as the mean value of NLR, as per our previous studies (18, 19).

The association between NLR and clinical-pathologic variables was assessed using the Chi-square test. The recurrence-free survival (RFS) and disease-specific survival (DSS) rates were calculated using the Kaplan-Meier method (the log rank test). The factors that were significant in the univariable analysis were then analyzed using the Cox proportional hazards method to determine the independent risk factors for RFS and DSS. All statistical analyses were performed using SPSS 20.0 and *p* < 0.05 was considered to be significant.

## Results

There were 123 patients (73 female and 50 male) in total: 25 patients from Affiliated Cancer Hospital, 58 patients from Affiliated Pediatric Hospital, and 40 patients from the Affiliated First Hospital. The mean age was 14.3 (range: 6–18) years. Thirteen patients had a history of blood malignancy. The mean time from initial blood malignancy to the diagnosis of parotid cancer was 8.4 (range: 6–12) years. The tumor stage of the patients was distributed as T1 in 50 patients, T2 in 43 patients, T3 in 15 patients, and T4 in 15 patients. Thirty-four patients underwent superficial parotidectomy, 15 patients underwent partial parotidectomy, and 74 patients underwent total parotidectomy. The facial nerve branches were sacrificed in 10 patients because of tumor invasion. Fine needle biopsy was conducted in 51 patients, and the pathology suspected a malignancy in 32 patients. Negative margins were achieved in 113 patients. A total of 39 patients underwent neck dissection, and positive neck disease was noted in 12 patients. The mean number of positive nodes was 1.2 (range: 1–4). Perineural invasion was noted in 12 patients, and lymphovascular invasion was noted in 9 patients (Table 1).

**Table 1:** Association between neutrophil-to-lymphocyte ratio and clinical pathologic variables.

**Variables**	**Neutrophil-to-lymphocyte ratio**		***p***
	** <2.51 (*n* = 68)**	**≥2.51 (*n* = 55)**	
**AGE**			
<14	16	12	
≥14	52	43	0.822
**SEX**			
Female	40	33	
Male	28	22	0.895
**TUMOR STAGE**			
T1+T2	58	35	
T3+T4	10	20	0.005
**NODE STAGE**			
N0	62	49	
N+	6	6	0.698
**DISEASE STAGE**			
I+II	49	33	
III+IV	19	22	0.158
**PERINEURAL INVASION**			
Positive	4	8	
Negative	64	47	0.133
**LYMPHOVASCULAR INVASION**			
Positive	3	6	
Negative	65	49	0.296
**INTRAPAROTID NODE METASTASIS**			
Positive	11	12	
Negative	37	40	0.985
**DISEASE GRADE**			
Low	60	40	
Intermediate+high	8	15	0.028
**MALIGNANCY HISTORY**			
Yes	5	8	
No	63	47	0.197

Mucoepidermoid carcinoma occurred in 72 patients, acinic cell cancer occurred in 24 patients, basal cell adenocarcinoma occurred in 10 patients, myoepithelial cancer occurred in 10 patients, and cystadenocarcinoma occurred in 7 patients (Table 2).

**Table 2:** Distribution of cancer sub-type in pediatric patients.

**Cancer sub-type**	**Number (%)**
**LOW GRADE (*****n*** **=** **100)**	
Mucoepidermoid cancer	59
Acinic cell cancer	24
Basal cell adenocarcinoma	10
Cystadenocarcinoma	7
**INTERMEDIATE GRADE (*****n*** **=** **18)**	
Mucoepidermoid cancer	8
Myoepithelial cancer	10
**HIGH GRADE (*****n*** **=** **5)**	
Mucoepidermoid cancer	5

Information regarding IPN was retrieved for 100 patients, and IPN metastasis was reported in 23 patients. The mean number of positive IPNs was 1.2 (range: 1–3), and the mean diameter of positive nodes was 0.9 (range: 0.4–2.1) cm.

The mean NLR was 2.51 and ranged from 1.7 to 6.1. The association between NLR and clinic-pathological variables are presented in Table 1; tumor stage and disease grade were significantly correlated with NLR (all *p* < 0.05).

**Figure 1:**
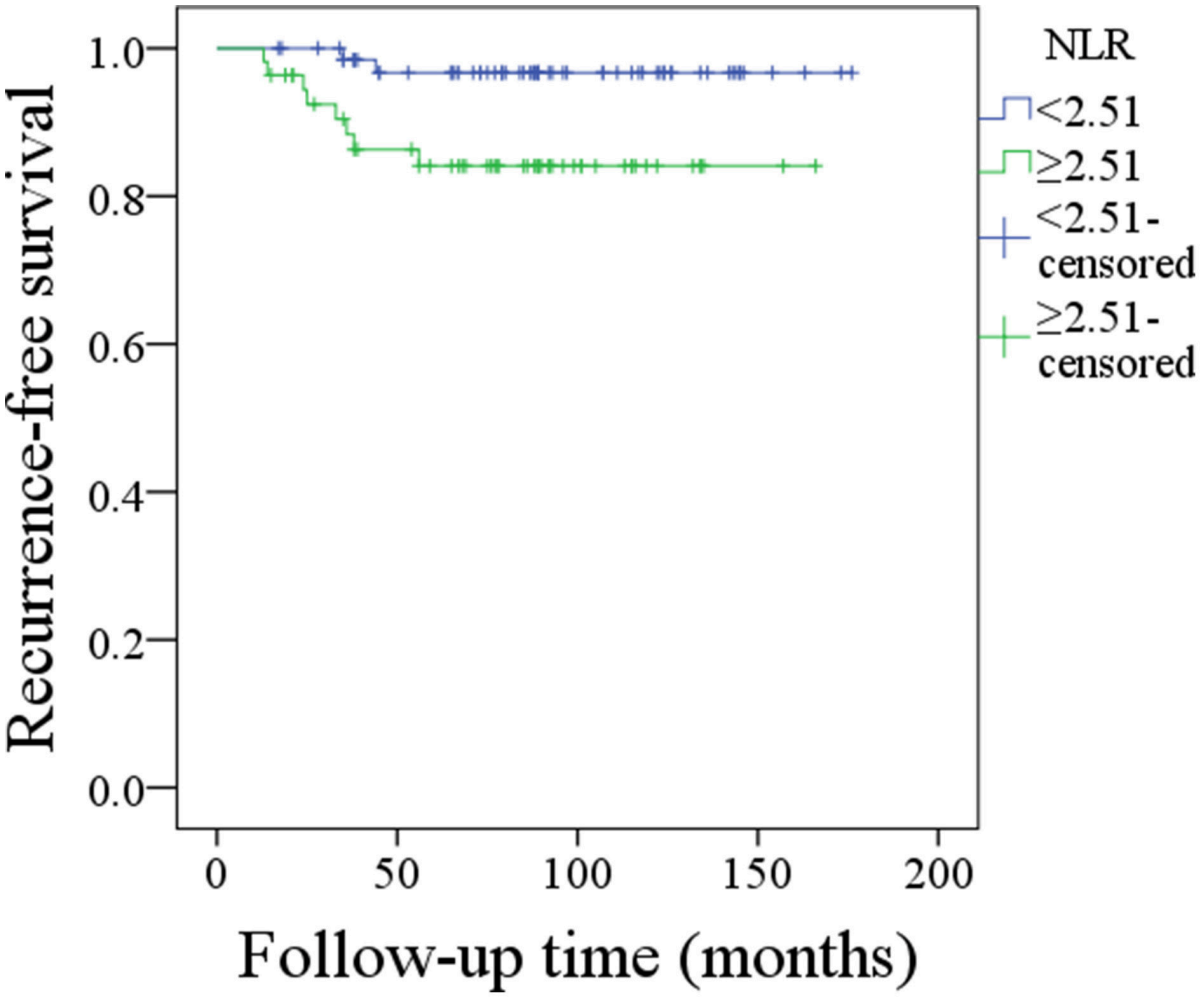
Recurrence-free survival in patients with different neutrophil-lymphocyte ratios (NLRs) (*p* = 0.016).

During follow-up, which had a mean duration of 88.1 months (range: 15–176) months, 36 patients underwent postoperative radiotherapy, and 8 patients also received chemotherapy. Recurrence was noted in 10 patients: 6 patients locally, 2 patients loco-regionally, 1 patient regionally, and 1 patient distantly. The 10-year RFS rate was 91%. In patients with NLR < 2.51, the 10-year RFS rate was 97%, and in patients with NLR ≥ 2.51, the 10-year RFS rate was 84%; this difference was significant (*p* = 0.016, Figure 1). Univariable analysis also found that tumor stage, disease grade, malignancy history, IPN metastasis, and resection extent were associated with recurrence; further Cox model analysis confirmed that disease grade, malignancy history, IPN metastasis, and NLR were independent prognostic factors (Table 3).

**Table 3:** Predictors for recurrence-free survival in pediatric patients with parotid cancer.

**Variables**	**Univariable analysis**	**Multivariable analysis**	
		**OR (95%CI)**	***p***
Age ( ≤ 14 vs. >14)	0.117		
Sex	0.329		
Nerve invasion	0.088		
Lymphovascular invasion	0.051		
Radiotherapy	0.162		
Margin status	0.059		
Node stage (cN0 vs. cN+)	0.088		
Tumor stage (T1+T2 vs. T3+T4)	0.031	2.136(0.956–7.311)	0.068
Grade (Low vs. intermediate+high)	0.002	2.222(1.288–5.145)	0.004
Malignancy history	0.011	1.342(1.017–2.687)	0.033
Intraparotid node metastasis	0.003	1.805(1.397–4.003)	0.003
Resection extent (TP vs. PP+SP^*^)	0.014	1.476(0.875–8.113)	0.245
NLR (<2.51 vs. ≥2.51)	0.016	1.169 (1.018–2.148)	0.011

* *TP, total parotidectomy; PP, partial parotidectomy; SP, superficial parotidectomy*.

**Figure 2:**
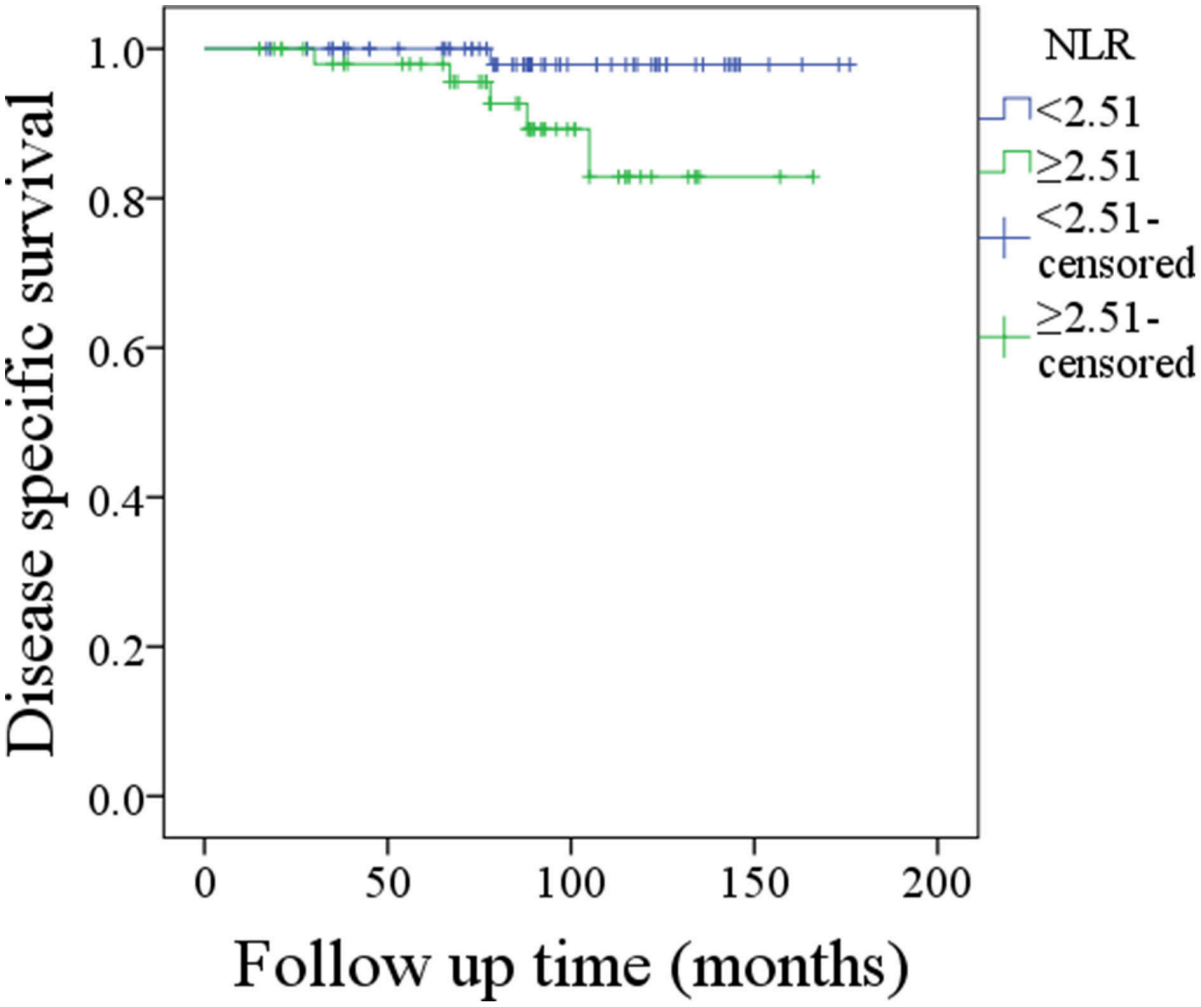
Disease-specific survival in patients with different neutrophil-lymphocyte ratios (NLRs) (*p* = 0.035).

A total of 6 patients died of the disease, and the 10-year DSS rate was 92%. In patients with NLR < 2.51, the 10-year DSS rate was 98%, and in patients with NLR ≥ 2.51, the 10-year DSS rate was 83%; this difference was also significant (p = 0.035, Figure 2). Univariable analysis found that tumor stage, disease grade, malignancy history, and IPN metastasis were associated with death; further Cox model analysis confirmed that disease grade, malignancy history, IPN metastasis, and NLR were independent prognostic factors (Table 4; Supplementary Material).

**Table 4:** Predictors for disease-specific survival in pediatric patients with parotid cancer.

**Variables**	**Univariable analysis**	**Multivariable analysis**	
		**OR (95%CI)**	***p***
Age ( ≤14 vs. >14)	0.336		
Sex	0.134		
Nerve invasion	0.054		
Lymphovascular invasion	0.091		
Radiotherapy	0.077		
Margin status	0.123		
Node stage (cN0 vs. cN+)	0.367		
Tumor stage (T1+T2 vs. T3+T4)	0.004	3.336(0.923–6.331)	0.064
Grade (Low vs. intermediate+high)	0.011	2.049(1.233–5.312)	0.002
Malignancy history	0.025	1.226(1.002–2.731)	0.022
Intraparotid node metastasis	<0.001	2.110(1.297–4.997)	0.001
Resection extent (TP vs. PP+SP^*^)	0.119		
NLR (<2.51 vs. ≥2.51)	0.035	1.448(1.012–3.231)	0.009

* *TP, total parotidectomy; PP, partial parotidectomy; SP, superficial parotidectomy*.

## Discussion

The significance of NLR had been evaluated only for inflammatory disorders, allergic conditions, and infectious diseases in pediatrics (20–22). Vasquez et al. (23) was the first to describe the prognostic role of NLR in pediatric sarcomas; the authors found that worse overall survival was strongly predicted by an NLR > 2, and there was a significant association between high NLR and poor histologic response as well as metastatic disease. The present study was the first to analyze how NLR affected survival in pediatric patients with parotid cancer, and we found that RFS and DSS rates were significantly decreased by high NLR in multivariable analysis. For adult patients, only two papers have assessed a similar question (16, 17). Damar et al. (16) found that, compared to patients with benign salivary tumors, NLR was significantly higher in patients with malignant salivary gland tumors, and NLR was significantly related to disease grade, which is similar to findings reported in the current study; moreover, it was noted that the higher NLR was associated with more advanced stages of disease. Kawakita et al. (17) found that, compared to baseline levels in patients with salivary duct carcinoma, an NLR > 2.5 meant there was nearly 2-fold risk of death.

The exact mechanism underling the associations between NLR and clinical-pathologic variables, as well as prognosis, remain unclear; based on previous evidence, there are some possible explanations. On the one hand, the status of the immune system and systemic inflammation is reflected by the pretreatment NLR. Elevation of neutrophils is a sign of local as well as systemic inflammatory responses. Neutrophils produce cytokines and angiogenic factors, and these agents play important roles in promoting tumor development; furthermore, hematological markers might be surrogate makers of cancer cachexia that are related to poor survival (24, 25). On the other hand, lymphocytes are related to immune surveillance and act by eliminating cancer cells (26). Therefore, a high NLR may predict worse prognosis.

The significance of IPN metastasis in pediatric parotid cancer has never been evaluated. We firstly reported that IPN metastasis was related to worse prognosis. Similar findings were previously reported in adult patients (24–30); Lim et al. (27) found that, compared with patients without IPN metastasis, patients with cN0 neck and IPN metastasis were more likely to develop locoregional recurrence. Klussmann et al. (28) noted that the involvement of IPN introduced additional significant risk for tumor recurrence in parotid cancer. Nisa et al. (29) described reported that decreased disease-free survival could be expected in patients with IPN metastasis. Therefore, IPN metastasis is related to higher risk of recurrence in both pediatric and adult medicine. This finding might be explained by the following: first, IPNs consist of superficial and deep parotid nodes; un-resected positive IPNs might be left after partial or superficial parotidectomy, and recurrence would be expected. Second, the N parameter in the TNM classification refers to regional, cervical lymph nodes, and IPN metastasis was not included in any of the groups of neck lymph nodes; thus, IPN appears to have some sentinel role for predicting neck disease.

Another interesting finding was that malignancy history predicts worse prognosis. Parotid cancer as a secondary malignancy in pediatrics had been presented by case reports (31); owing to its extreme rarity, the prognosis of these patients remains unclear. Védrine et al. (5) performed a study of maximum sample size of secondary parotid cancer; a total of 18 mucoepidermoid cancer patients were included, and 11 patients were defined as having secondary disease. There was no difference in distribution according to sex, age, tumor location or tumor grade; however, the distribution based on the clinical stage (stages 1–2 vs. stages 3–4) did differ, and there was less advanced clinical stage in the group with mucoepidermoid carcinoma as secondary disease. Nevertheless, the differences in overall survival, DSS, and disease-free survival between the two groups were not statistically significant. It was noted patients with histories of malignancy had worse prognoses. A possible explanation might be that previous chemotherapy for blood malignancy had a significantly adverse impact on the lymph defense system, and this impact negatively decreased the prognosis.

The limitations of the current study must be acknowledged, as follows: first, this is a retrospective study, and there is inherent bias that might decease the statistical power; second, it should be recognized that neutrophils and lymphocytes counts are nonspecific parameters because they could be influenced by concomitant conditions, such as infections or inflammation; and third, the pathology sections were examined by different pathologists in the three hospitals, and due to the differences in diagnostic ability, there might have been undetected IPNs.

## Conclusions

In summary, pretreatment NLR is significantly associated with survival in pediatric patients with parotid cancer.

## Data Availability

All data generated or analyzed during this study are included in this published article. The primary data may be obtained from the corresponding author.

## Ethics Statement

The Zhengzhou University institutional research committee approved our study and all participants signed informed consent agreements. All related procedures were consistent with Ethics Committee regulations.

## Author Contributions

SL, PL, FL, and QF: study design and manuscript writing. DS, SL, PL, and QF: studies selecting and data analysis and manuscript revising. SL, PL, and QF: study quality evaluating. All authors have read and approved the final manuscript.

### Conflict of Interest Statement

The authors declare that the research was conducted in the absence of any commercial or financial relationships that could be construed as a potential conflict of interest.

## References

[1] Dombrowski ND Wolter NE Irace AL Cunningham MJ Mack JW Marcus KJ. Mucoepidermoid carcinoma of the head and neck in children. Int J Pediatr Otorhinolaryngol. (2019) 120:93–9 10.1016/j.ijporl.2019.02.020 30772619

[2] Zamani M Grønhøj C Schmidt Jensen J von Buchwald C Charabi BW Hjuler T. Survival and characteristics of pediatric salivary gland cancer: a systematic review and meta-analysis. Pediatr Blood Cancer. (2019) 66:e27543. 10.1002/pbc.27543 30378272

[3] Fang QG Shi S Li ZN Zhang X Liu FY Sun CF. Epithelial salivary gland tumors in children: a twenty-five-year experience of 122 patients. Int J Pediatr Otorhinolaryngol. (2013) 77:1252–4. 10.1016/j.ijporl.2013.04.034 23746416

[4] Janz TA Camilon PR Nguyen SA Levi JR Lentsch EJ. Has the management of pediatric mucoepidermoid carcinoma of the parotid gland changed? Laryngoscope. (2018) 128:2408–14. 10.1002/lary.27192 29658113

[5] Védrine PO Coffinet L Temam S Montagne K Lapeyre M Oberlin O. Mucoepidermoid carcinoma of salivary glands in the pediatric age group: 18 clinical cases, including 11 second malignant neoplasms. Head Neck. (2006) 28:827–33. 10.1002/hed.20429 16783829

[6] Carlson ER Schlieve T. Salivary gland malignancies. Oral Maxillofac Surg Clin North Am. (2019) 31:125–44. 10.1016/j.coms.2018.08.007 30449524

[7] Qureshi SS Bhagat M Singhal N Tathe N Kembhavi S Laskar S. Clinical characteristics and treatment outcomes of primary and recurrent malignancy involving the salivary glands in children. Head Neck. (2016) 38:852–6. 10.1002/hed.24114 25917761

[8] Radomski S Dermody S Harley EHJr. Clinical characteristics and outcomes of major salivary gland malignancies in children. Laryngoscope. (2018) 128:1126–32. 10.1002/lary.26946 28990673

[9] Rebours C Couloigner V Galmiche L Casiraghi O Badoual C Boudjemaa S. Pediatric salivary gland carcinomas: diagnostic and therapeutic management. Laryngoscope. (2017) 127:140–7. 10.1002/lary.26204 27497071

[10] Xu B Aneja A Ghossein R Katabi N. Salivary gland epithelial neoplasms in pediatric population: a single-institute experience with a focus on the histologic spectrum and clinical outcome. Hum Pathol. (2017) 67:37–44. 10.1016/j.humpath.2017.07.007 28739497

[11] Kulbe H Chakravarty P Leinster DA Charles KA Kwong J Thompson RG. A dynamic inflammatory cytokine network in the human ovarian cancer microenvironment. Cancer Res. (2012) 72:66–75. 10.1158/0008-5472.CAN-11-2178 22065722 PMC3252703

[12] Ashok A Keener R Rubenstein M Stookey S Bajpai S Hicks J. Consequences of interleukin 1β-triggered chronic inflammation in the mouse prostate gland: altered architecture associated with prolonged CD4+ infiltration mimics human proliferative inflammatory atrophy. Prostate. (2019) 79:732–74. 10.1002/pros.23784 30900284

[13] Peinado H Lavotshkin S Lyden D. The secreted factors responsible for pre-metastatic niche formation: old sayings and new thoughts. Semin Cancer Biol. (2011) 21:139–46. 10.1016/j.semcancer.2011.01.002 21251983

[14] Kano S Homma A Hatakeyama H Mizumachi T Sakashita T Kakizaki T. Pretreatment lymphocyte-to-monocyte ratio as an independent prognostic factor for head and neck cancer. Head Neck. (2017) 39:247–53. 10.1002/hed.24576 27617428

[15] Kapoor S. Neutrophil to lymphocyte ratio and its association with tumor prognosis in systemic malignancies. J Surg Oncol. (2013) 107:560. 10.1002/jso.23291 23192308

[16] Damar M Dinç AE Erdem D Aydil U Kizil Y Eravci FC Pretreatment neutrophil-lymphocyte ratio in salivary gland tumors is associated with malignancy. Otolaryngol Head Neck Surg. (2016) 155:988–96. 10.1177/0194599816659257 27436419

[17] Kawakita D Tada Y Imanishi Y Beppu S Tsukahara K Kano S. Impact of hematological inflammatory markers on clinical outcome in patients with salivary duct carcinoma: a multi-institutional study in Japan. Oncotarget. (2017) 8:1083–91. 10.18632/oncotarget.13565 27894101 PMC5352036

[18] Fang Q Liu F Seng D. Oncologic outcome of parotid mucoepidermoid carcinoma in pediatric patients. Cancer Manag Res. (2019) 11:1081–5. 10.2147/CMAR.S192788 30774436 PMC6357880

[19] Fang Q Li P Qi J Luo R Chen D Zhang X. Value of lingual lymph node metastasis in patients with squamous cell carcinoma of the tongue. Laryngoscope. (2019). 10.1002/lary.27927. [Epub ahead of print]. 30861130

[20] Aktar F Tekin R Bektas MS. Diagnostic role of inflammatory markers in pediatric *Brucella arthritis*. Ital J Pediatr. (2016) 42:3. 10.1186/s13052-016-0211-5 26753565 PMC4709903

[21] Dogru M Yesiltepe Mutlu RG. The evaluation of neutrophil-lymphocyte ratio in children with asthma. Allergol Immunopathol. (2016) 44:292–6. 10.1016/j.aller.2015.09.005 26777420

[22] Eryilmaz A Basal Y Tosun A. The neutrophil to lymphocyte ratios of our pediatric patients with Bell's palsy. Int J Pediatr Otorhinolaryngol. (2015) 79:2374–7. 10.1016/j.ijporl.2015.10.047 26602556

[23] Vasquez L León E Beltran B Maza I Oscanoa M Geronimo J. Pretreatment neutrophil-to-lymphocyte ratio and lymphocyte recovery: independent prognostic factors for survival in pediatric sarcomas. J Pediatr Hematol Oncol. (2017) 39:538–46. 10.1097/MPH.0000000000000911 28697168

[24] Hagerling C Werb Z. Neutrophils: critical components in experimental animal models of cancer. Semin Immunol. (2016) 28:197–204. 10.1016/j.smim.2016.02.003 26976824 PMC4934898

[25] Liu F Cheng GY Fang QG Sun Q. Natural history of untreated squamous cell carcinoma of the head and neck. Clin Otolaryngol. (2018) 44:200–3. 10.1111/coa.13260 30444304

[26] Mohammed ZM Going JJ Edwards J Elsberger B Doughty JC McMillan DC. The relationship between components of tumour inflammatory cell infiltrate and clinicopathological factors and survival in patients with primary operable invasive ductal breast cancer. Br J Cancer. (2012) 107:864–73. 10.1038/bjc.2012.347 22878371 PMC3426752

[27] Lim CM Gilbert MR Johnson JT Kim S. Clinical significance of intraparotid lymph node metastasis in primary parotid cancer. Head Neck. (2014) 36:1634–7. 10.1002/hed.23507 24123567

[28] Klussmann JP Ponert T Mueller RP Dienes HP Guntinas-Lichius O. Patterns of lymph node spread and its influence on outcome in resectable parotid cancer. Eur J Surg Oncol. (2008) 34:932–7. 10.1016/j.ejso.2008.02.004 18358679

[29] Nisa L Salmina C Dettmer MS Arnold A Aebersold DM Borner U. Implications of intraglandular lymph node metastases in primary carcinomas of the parotid gland. Laryngoscope. (2015) 125:2099–106. 10.1002/lary.25342 25946394

[30] Feng YP Liu F Cheng GY Fang QG Niu XY He W. Significance of intraparotid node metastasis in predicting local control in primary parotid cancer. Laryngoscope. (2018). 10.1002/lary.27701. [Epub ahead of print]. 30549298

[31] Tugcu D Akici F Aydogan G Salcioglu Z Akcay A Sen H. Mucoepidermoid carcinoma of the parotid gland in childhood survivor of acute lymphoblastic leukemia with need of radiotherapy for treatment and review of the literature. Pediatr Hematol Oncol. (2012) 29:380–5. 10.3109/08880018.2012.673696 22568803

