# Supplementary material for: Prognostic Value of the Pretreatment Neutrophil-to-Lymphocyte Ratio in Pediatric Parotid Cancer
Source: Front Pediatr. 2019 May 24;7:207. doi: 10.3389/fped.2019.00207 (PMC6543004; doi:10.3389/fped.2019.00207)
Supplement: Supplementary file 1 [file Table_1.DOC]

Supplied table

Significance of NLR in specific parotid cancer

|  | RFS | | DSS | |
| --- | --- | --- | --- | --- |
|  | Univariable | Multivariable | Univariable | Multivariable |
| Mucoepidermoid cancer (n=72) | <0.001 | <0.001 | <0.001 | <0.001 |
| Acinic cell cancer (n=24) | 0.034 | 0.085 | 0.048 | 0.041 |
| Basal cell adenocarcinoma (n=10) | 0.245 |  | 0.325 |  |
| Cystadenocarcinoma (n=7) | 0.574 |  | 0.212 |  |
| Myoepithelial cancer (n=10) | 0.334 |  | 0.178 |  |
